# Comparison of French training and non-training general practices: a cross-sectional study

**DOI:** 10.1186/s12909-016-0649-6

**Published:** 2016-04-27

**Authors:** Laurent Letrilliart, Pauline Rigault-Fossier, Benoit Fossier, Nadir Kellou, Françoise Paumier, Christophe Bois, Stéphanie Polazzi, Anne-Marie Schott, Yves Zerbib

**Affiliations:** Universite de Lyon 1, Collège universitaire de médecine générale, Lyon, France; Universite de Lyon, Equipe d’Accueil HESPER 7425, Lyon, France; Département de médicine générale, Universite de Grenoble, Grenoble, France; Département de médicine générale, Universite de Saint-Etienne, Saint-Etienne, France; Hospices Civils de Lyon, Pôle IMER, Lyon, France; Universite de Lyon 1, Equipe d’Accueil Sciences et Société, Historicité, Education et Pratique (S2HEP) 4148, Lyon, France

**Keywords:** French, General practice, Performance, Representativeness, Training practices

## Abstract

**Background:**

As the medicine practiced in hospital settings has become more specialized, training in primary care is becoming increasingly essential for medical students, especially for future general practitioners (GPs). Only a few limited studies have investigated the representativeness of medical practices delivering this training. The aim of this study was to assess the representativeness of French GP trainers in terms of socio-demographics, patients and activities.

**Methods:**

We conducted a cross-sectional study covering all private GPs practicing in the Rhône-Alpes region of France in 2011. This population consisted of 4992 GPs, including 623 trainers and 4369 non-trainers, managing 8,198,684 individual patients. Data from 2011 to 2012 were provided by the Regional Health Care Insurance (RHCI). We compared GP trainers with non-trainers using the Pearson chi-square test for qualitative variables and the Student *t*-test for quantitative variables

**Results:**

GP trainers do not differ from non-trainers for gender, but they tend to be younger, more frequently in mid-career, and more likely to practice in a rural area. Their patients are broadly representative of patients attending general practice for age (with the exception of a higher consultation rate for infants), but patients with medical fee exemption status relating to low income are underrepresented. GP trainers have a heavier workload in terms of office visits and on-call duties. They prescribe a higher proportion of generic drugs, perform more electrocardiograms and cervical smears, and fewer plaster casts. GP trainers show better performance in diabetes follow-up, and to a lesser extent for seasonal flu vaccination and mammograms.

**Conclusions:**

GPs and patients of training practices are globally representative, which is particularly critical in countries such as France, where the length of specialty training in a general practice setting is still limited to a few months. In addition, GP trainers tend to have better clinical performance, which conforms to their teaching modelling role and may encourage other GPs to become trainers.

## Background

In recent years, general practice has been increasingly recognized as a scientific discipline at universities worldwide [1]. This movement has been associated with the increasing involvement of general practitioners (GPs) in training and research, albeit to varying extents depending on the country [2, 3]. In France, general practice has become a medical specialty since the creation of a specific general practice degree in 2004. As the medicine practiced in hospital settings becomes more specialized, training in primary care is increasingly essential for medical students, especially for future GPs [4]. The 6 years undergraduate curriculum includes mandatory training in a general practice setting, which lasts 1.5 months (full-time equivalent). The 3 year postgraduate curriculum includes 2 years of training in the hospital setting (with a mandatory 6 month period at a university hospital), and 6 to 12 months at a GP surgery.

The representativeness of GP trainers is a challenge for medical education in primary care, as it supports the exposure of medical students to a large and appropriate patient-mix. Along with the quality of supervision, the patient mix is considered as a crucial factor for learning in general practice [5, 6]. Indeed, training in GP surgeries must be diverse enough to allow students to encounter the various health problems that are managed, and care procedures performed in primary care [7]. Only a few studies have investigated the representativeness of practices delivering training. Three European [8–10] and one Australian [11] contemporary studies showed differences between trainers and non-trainers in their socio-demographic characteristics or their activities. These studies were limited either in the number of physicians investigated or in the number of characteristics compared.

The aim of our survey was therefore to study the representativeness of GP trainers in terms of socio-demographics, patients and activities.

## Methods

### Sampling

We conducted a cross-sectional study, entitled the REGE study (REprésentativité des Généralistes Enseignants), including all private GPs in the French Rhone-Alpes region.

In 2011, 5815 GPs were listed as registered by the Regional Health Care Insurance (RHCI). The 738 GPs involved in teaching or training medical students were identified from lists obtained from the departments of general practice at the Universities of Lyon, Grenoble and Saint-Etienne. We provided these lists to the RHCI, after excluding 15 GPs because they refused to participate (*n* = 4) or because they were teachers at the university but not trainers at their surgery (*n* = 11). Among GP trainers, 52 were not registered in the RHCI database and were not included in the study.

Physicians who were qualified as GPs, but with a special clinical specialism (homeopathy, acupuncture, nutrition, sport medicine, vascular medicine, etc.), were excluded (48 trainers and 600 non-trainers). GPs without any reimbursed consultation in 2011 or 2012 were considered as inactive and therefore excluded (*n* = 180).

**Fig. 1:**
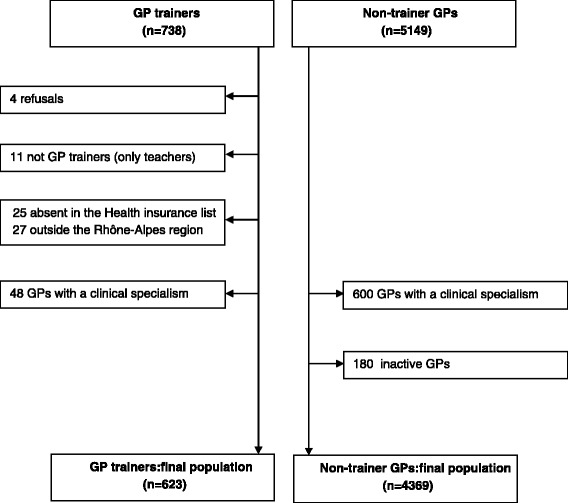
Flow chart presenting the two populations under study

The final population consisted of 4992 GPs, including 623 trainers and 4369 non-trainers (Fig. 1).

### Data collection

Apart from the training status of GPs, all data were provided by the RHCI. They referred to the full year 2011 or 2012, depending on the type of data.

GPs’ socio-demographic characteristics included gender, age, year of commencement of general practice, practice location, and department. GPs’ practice characteristics included patients’ age (global distribution and proportion of consultations with infants or children) and medical fee exemption status (full financial coverage by the national public health care insurance, for long-term conditions or for low income). GPs’ activities included: the number of individual patients consulted per year; the number of office and home visits; participation in on-call duties; prescription of reimbursed drugs and of sick leave days with allowances; the provision of four medical procedures (electrocardiogram, suture, plaster cast, cervical smear); and three performance indicators (for seasonal flu vaccination, mammogram, glycated hemoglobin).

### Statistical analysis

We compared GP trainers with non-trainers, using the Pearson Chi-square test to compare proportions and the Student’s *t*-test to compare means. We assumed a significance level of .05.

## Results

In total, we investigated 4992 GPs and their 8,198,684 individual patients. Of the GPs, 623 (12.5 %) were trainers: either of undergraduate students (143) or of postgraduate students with various levels of autonomy (554). Postgraduate students on placement were either under direct (*n* = 410) or indirect (*n* = 251) supervision.

### GP sociodemographics (Table 1)

**Table 1:** Comparison of GP trainers and non-trainers according to their sociodemographics (year 2011)

	GP trainers		Non-trainer GPs		*p*-value
	(*n* = 623)		(*n* = 4369)		
	n	(%)	n	(%)	
Gender					0.38
Male	431	(69.2 %)	2945	(67.4 %)	
Female	192	(30.8 %)	1424	(32.6 %)	
Age (yrs.)					<10^−4^
≤ 36	62	(9.9 %)	405	(9.3 %)	
37–46	116	(18.6 %)	870	(19.9 %)	
47–56	277	(44.5 %)	1573	(36.0 %)	
≥ 57	168	(27.0 %)	1521	(34.8 %)	
Years in general practice					<10^−4^
≤ 11	178	(28.6 %)	1482	(33.9 %)	
12–21	159	(25.5 %)	996	(22.8 %)	
22–26	139	(22.3 %)	660	(15.1 %)	
≥ 27	147	(23.6 %)	1231	(28.2 %)	
Practice location					<10^−4^
Urban area	391	(62.8 %)	3080	(70.5 %)	
Rural area	232	(37.2 %)	1289	(29.5 %)	
Department					<10^−4^
Ain	42	(6.7 %)	330	(7.6 %)	
Ardèche	30	(4.8 %)	199	(4.6 %)	
Drôme	48	(7.7 %)	348	(8.0 %)	
Isère	125	(20.0 %)	825	(18.9 %)	
Loire	135	(21.7 %)	449	(10.2 %)	
Rhône	121	(19.4 %)	1293	(29.6 %)	
Savoie	49	(7.9 %)	356	(8.1 %)	
Haute-Savoie	73	(11.7 %)	569	(13.0 %)	

The gender distribution was similar between trainers and non-trainers. GP trainers were more likely to be aged between 47 and 56 years old (44.5 % vs. 36.0 %) and less likely to be aged over 57 years (27.0 % vs. 34.8 %) than non–trainers. They were more likely to have from 12 to 26 years of prior practice (47.8. % vs. 37.9 %), and less likely to have fewer than 12 years or more than 27 years of experience, than non-trainers. Trainers were more likely to work in a rural area than non-trainers (37.2 % vs. 29.5 %). They were more likely to practice in the Loire (21.7 % vs. 10.2 %) and less likely to practice in the Rhône department (19.4 % vs. 29.6 %) than non-trainers.

### GP practice characteristics (Table 2)

**Table 2:** Comparison of GP trainers and non-trainers according to their patients’ characteristics (year 2011)

	GP trainers		Non-trainer GPs		*p*-value
	(*n* = 623)		(*n* = 4369)		
	n	(%)	n	(%)	
Age					
By single patients^a^ (yrs.)					<10^−4^
< 16	262989	(24.4)	1644622	(23.1)	
16–59	582955	(54.1)	3958953	(55.6)	
60–69	101271	(9.4)	679921	(9.5)	
≥ 70	130194	(12.1)	837779	(11.8)	
By visits^b^ (yrs.)					<10^−4^
< 2	136014	(4.7)	767483	(4.1)	
2–6	149097	(5.2)	924461	(4.9)	
> 6	2598105	(90.1)	17213737	(91.0)	
Medical fee exemption status					
For long-term conditions^a^					<10^−4^
Yes	246005	(22.8)	1646616	(23.1)	
No	831404	(77.2)	5474659	(76.9)	
For low income^a^					<10^−4^
Yes	63512	(5.9)	481078	(6.8)	
No	1013897	(94.1)	6640197	(93.2)	

^a^Data are presented as numbers of single patients consulted during the year (*n* = 1077409 for GP trainers and *n* = 7121275 for non-trainers)

^b^Data are presented as numbers of visits during the year (*n* = 2883216 for GP trainers and *n* = 18905681 for non-trainers). These include repeated visits for a single patient

GP trainers managed slightly more (individual) patients younger than 16 years old (24.4 % vs. 23.1 %) or aged 70 or over (12.1 % vs. 11.8 %) than non-trainers. Trainers provided a higher proportion of consultations to infants (4.7 % vs. 4.1 %) and to children (5.2 % vs. 4.9 %) than non-trainers. They saw slightly fewer (individual) patients with an exemption status for a long-term condition (22.8 % vs. 23.1 %) and fewer for low income (5.9 % vs. 6.8 %) than non-trainers.

### GP activities (Table 3)

**Table 3:** Comparison of GP trainers and non-trainers according to their activities (year 2011 or 2012)

	GP trainers		Non-trainer GPs		*p*-value
	(*n* = 623)		(*n* = 4369)		
	m	(SD)	m	(SD)	
Individual patients consulted (year 2011)	1729	(556)	1630	(837)	0.004
Visits (year 2011)					
Office visits					
Global	4351	(1474)	4066	(2144)	0.001
Per patient	2.6	(0.6)	2.6	(1.1)	0.48
Home visits					
Global	277	(260)	262	(348)	0.28
Per patient	0.2	(0.1)	0.2	(0.2)	0.36
On-call duties [n (%)]					<10^−4^
Yes	448	(71.9)	2535	(58,0)	
No	175	(28.1)	1834	(42.0)	
Prescriptions (year 2011)					
Reimbursed drugs					
Global (Euros)	271339	(150250)	251745	(173100)	0.01
Per patient (Euros)	161.2	(86.1)	159.9	(89.1)	0.72
Proportion of generics	23.4 %	(5.7 %)	21.2 %	(6.0 %)	<10^−4^
Allowances for sick leave					
Global (days)	3815	(2350)	3828	(3157)	0.92
Medical procedures (year 2012)					
Electrocardiograms	36.9	(43.0)	21.1	(52.9)	<10^−4^
Sutures	3.7	(7.3)	4.6	(25.9)	0.39
Plaster casts	1.0	(4.0)	3.4	(28.8)	0.04
Cervical smears	1.2	(3.5)	0.4	(2.6)	<10^−4^
Performance indicators^a^					
Seasonal flu vaccination^b^ (year 2012) [n (%)]					<10^−4^
Yes	46469	(54.5)	268706	(52.6)	
No	38856	(45.5)	242377	(47.4)	
Mammogram^c^ (years 2011–2012) [n (%)]					<10^−4^
Yes	43746	(65.5)	259666	(63.1)	
No	23079	(34.5)	151698	(36.9)	
Three or four glycated hemoglobin assays a year^d^ (year 2012) [n (%)]					<10^−4^
Yes	14040	(60.6)	79800	(53.8)	
No	9126	(39.4)	68415	(46.2)	

^a^The three indicators refer to the percentage of patients registered with the GP who have had the procedure during the recommended time period

^b^The target population consisted of patients aged 65 years or over in the patient list (*n* = 85325 for GP trainers and *n* = 511083 for non-trainers)

^c^The target population consisted of female patients aged 50 to 74 years in the patient list (*n* = 66825 for GP trainers and *n* = 411364 for non-trainers)

^d^The target population consisted of patients with diabetes in the patient list (*n* = 23166 for GP trainers and *n* = 148215 for non-trainers)

On average, GP trainers managed more (single) patients than non-trainers (1729 vs. 1630), with a higher number of office visits (4351 vs. 4066), but achieved the same number of office and home visits per patient. Trainers participated more in on-call duties than non-trainers (71.9 % vs. 58.0 %). They prescribed a higher proportion of generic drugs compared to non-trainers (23.4 % vs. 21.2 %). In 2012, trainers performed more electrocardiograms (36.9 vs. 21.1) and cervical smears (1.2 vs.0.4), and fewer plaster casts (1.0 vs. 3.4), than non-trainers. They achieved better coverage for seasonal flu vaccination (54.5 % vs. 52.6 %) and a higher rate of mammograms (65.5 % vs. 63.1 %) in the respective target populations, and a more regular follow-up of glycated hemoglobin (60.6 % vs. 53.8 %) in patients with diabetes, than non-trainers.

## Discussion

According to our comprehensive data from the Rhône-Alpes region of France, GP trainers are representative of GPs for gender, but they are younger, more frequently in mid-career, and more likely to be practicing in a rural area. Their patients are broadly representative of patients attending general practice for age and for exemption status relating to long-term conditions. GP trainers have a heavier workload in terms of office visits and on-call duties. They prescribe more generic drugs, and perform more electrocardiograms and cervical smears, but fewer plasters. GP trainers show better clinical performance in diabetes follow-up, and to a lesser extent for seasonal flu vaccination and mammograms.

### Strengths and weaknesses

The database we used covered a large population of GPs, and no data was missing. Rhône-Alpes is the second most populated French region, and GP trainers account for 12.5 % of all GPs in this region, as compared to 10.5 % at the national level [12]. We were unable to study some important variables like patient gender or GP weekly time in clinical practice. We were only able to collect a few performance indicators which can be considered as proxies for quality of care [13]. The value of performing mammograms is questionable, moreover, as they can be prescribed either for screening (mainly) or for diagnostic purposes, and as breast cancer screening is increasingly debated internationally [14].

We excluded the GPs registered in the RCHI database as having a special clinical specialism because their specialized activity was their main activity. We observed, indeed, that all but one of them (48 trainers and 599 non-trainers) did not perform any of the four medical procedures analyzed and had none of the three performance indicators available. Presumably, both trainer and non-trainer samples included GPs having only part-time specialized clinical activity, but we could not identify them in this study.

Some of the GP trainers (40.3 %) provide indirect supervision to their trainees, which means that the trainees practice autonomously [15]. This type of training may influence the health problems managed during the visits [16, 17]. However, we observed similar trends for performance indicators after exclusion of these particular GP trainers (data not presented).

This study explored parts of five core competencies required for general practice out of the six defined by Wonca Europe, namely: primary care management, person-centred care, specific problems solving skills, comprehensive approach and community orientation [18]. We did not investigate some important parts of these competencies, like care coordination, doctor-patient relationship or frequency of health problems, and did not investigate the holistic approach competency at all. Regarding the doctor-patient relationship, a recent study in England stressed that GP training practices offer more patient-centred care than non-training practices, which remains to be assessed in France [19].

### Global GP trainers and patients representativeness

In France, GP trainers are younger than non-trainers, as observed in Germany and Australia, but contrary to what has been observed in the Netherlands [8, 10, 11]. Although, in France, 1 to 3 years of practice are required before becoming a trainer, we can suppose that the younger generation of GPs is more interested in teaching, having received training in general practice. The representativeness of GP trainers in terms of gender has also been observed in German GPs but not in Dutch GPs, who are more often male [8, 10]. Trainees need to be exposed to gender-specific health problems and care procedures. As gender distribution within patient lists is influenced by GP gender [20], trainees should ideally be trained both by male and female trainers. In any case, an increasing female-to-male ratio is expected for trainers and non-trainer GPs in the future [21]. French GP trainers are more likely to be working in rural areas, as are Australian GP trainers [11]. This observation is consistent with the underrepresentation of GP trainers in the Rhône department, which is a rather urban area including the city of Lyon [22]. Rural trainers may help to reduce rural shortages in primary care, as medical students who have experienced rural training, either at undergraduate or postgraduate level, are more likely to become rural GPs [23, 24]. In addition, trainees can usually learn a greater range of procedural skills in rural than in urban practices [25]. In Australia, more rural regional practices have more GPs (usually five or more) [11], which may be a factor in the motivation to teach and in providing quality supervision. This organization seems partly different in France, with more trainers but smaller practices in more rural areas [26]. Patients attending GP trainers are globally representative in terms of age. In particular, this ensures that medical students are trained specifically in the management of elderly, who represent an increasing proportion of general practice patients [27]. The higher consultation rate for infants ensures that trainees learn about their specific healthcare needs [28]. The relative representativeness of patients with long-term conditions is reassuring, as chronic care represents a critical issue in primary care [29]. However, for the trainees under indirect supervision, the small difference between trainers and non-trainers observed in our study may add to the the lack of exposure due to patients with chronic conditions preferring to be managed by their usual practitioner [30].

The underrepresentation of patients with exemption status for low income may be due to a lower density of teaching practices in socially deprived areas, as observed in the Rhône department and in the UK [31, 32]. This issue should be further explored, as trainees should learn to care for deprived patients, who present with specific expectations and health problems [33, 34].

### A trend toward better clinical performance

GP trainers have higher scores than non-trainers according to the preventive care data collected, i.e. flu vaccination, mammograms and diabetes follow-up. An English study also found that GP trainers perform better in infant vaccination, cervical smears and asthma management [9]. In a Dutch study, GP trainers had better scores for management of diabetes and cardiovascular diseases [8]. More generally, training practices are associated with higher scores in the British Quality and Outcomes Framework [31]. Several factors may explain this better performance. Firstly, GP trainers are encouraged by interacting with their students to immerse themselves back into the basics of clinical medicine, and to read medical literature [35]. In addition, they tend to have a higher level of academic qualification and to attend more continuous professional development sessions, than non-trainers [8, 36]. Finally, training practices tend to be more innovative, as observed in England, in terms of screening services, practice organization and health record systems [37]. Performance in preventive medicine, including screening, seems to be particularly valued by students [38].

GP trainers perform more, albeit few, electrocardiograms and cervical smears than non-trainers, as in England and the Netherlands [7, 8]. These activities may be facilitated by better diagnostic equipment in training practices [8, 31]. In our study, the generic substitution rate is barely higher for GP trainers, even if it remains low compared to other European countries [39]. GP trainers have a critical role in this respect, as French university hospitals still predominantly prescribe brand-name drugs [40].

The higher number of consultations for GP trainers in our study is consistent with findings from the Netherlands [8]. Conversely, English GP trainers proved to have a lower workload, which is due to smaller patient lists compared to non-trainers, and possibly encouraged by compensatory payments dedicated to teaching [7, 41]. Quality of care is likely to be associated with the length of consultation. In particular, doctors who provide longer consultations tend to deliver more lifestyle advice and preventive activities [42]. Although consultations with GP trainers may have a slightly different sequencing, there is no evidence that they are longer than consultations in non-training practices [43, 44]. The lack of data on weekly time in clinical practice did not allow us to compare the mean duration of visits. Trainers carry out much more on-call duties, allowing medical students to learn to manage unplanned care.

### Stakes for education

A balance has to be reached between the need for recruiting a large workforce of GP trainers and the quality of the training of the medical students. The effectiveness of this training depends on the range of attending patients and the quality of students’ supervision [45]. The European Academy of Teachers in General Practice (EURACT) has not proposed any specific criteria regarding the size and variety of the patient list expected from a GP trainer [46]. The National College of French GPs recommends that trainers provide between 2500 and 7000 consultations in a year [13]. However, such a criterion cannot be extrapolated to any country, as the number and the length of consultations depend on the health care system [47]. According to our data, only 53 GP trainers (8.5 %) manage fewer than 2500 consultations and 27 (4.3 %) more than 7000 in the Rhône-Alpes region. GP trainers generally seem to provide a relatively appropriate patient mix to their students, at least in terms of age and chronic conditions. The EURACT and the National College of French GPs provide only loose recommendations on the educational competencies required from GP trainers. A Dutch group has proposed a set of criteria, based on teaching attitude, knowledge and skills, and personality traits [48]. The assessment of these competencies of GP trainers was out of the scope of our study.

## Conclusion

GPs and patients of training practices are globally representative of other GPs and patients. Such exposure of medical students to a large and appropriate patient mix is particularly critical in countries such as France, where the length of specialty training in a general practice setting is still limited to a few months. In addition, GP trainers tend to have better clinical performance, which conforms to their teaching modelling role and may encourage other GPs to become trainers.

As far as more and more general practices are expected to provide training to undergraduate and postgraduate students of various levels, criteria are needed for the recruitment of GP trainers. In addition to their educational competencies, incorporating clinical and communication abilities, these criteria should include an appropriate size and variety of their patient list to best fit the students’ learning needs and should be adapted to the curriculum in each country. National and international organizations of teachers in general practice should provide recommendations on the profile required from GP trainers, to be adapted secondarily to any local context.

The initial selection of training practices should be complemented afterwards by a quality assessment and improvement process, based in particular on the satisfaction levels of trainees and patients. More research is thus needed on how to define and assess the complex issues involved in the provision of high-quality GP training.

### Ethics approval and consent to participate

All the data provided by the RHCI were fully anonymized. This study was covered by a general agreement obtained from the French Committee for Informatics and Freedom (CNIL, record number 1639624). It was also declared to the Committee for the Protection of Persons (CPP Sud-Est II, record number 2013-025-1).

### Consent for publication

Not applicable.

### Availability of data and materials

Please contact the first author for accessing the database used in this study.
